# Oxidative stress and antioxidant response in fibroblasts from Werner and Atypical Werner Syndromes

**DOI:** 10.18632/aging.100649

**Published:** 2014-03-31

**Authors:** Marta Seco-Cervera, Marta Spis, José Luis García-Giménez, José Santiago Ibañez-Cabellos, Ana Velázquez-Ledesma, Isabel Esmorís, Sergio Bañuls, Giselle Pérez-Machado, Federico V Pallardó

**Affiliations:** ^1^ CIBERER. Centro de Investigación Biomédica en Red de Enfermedades Raras, Valencia, Spain; ^2^ FIHCUV-INCLIVA. Valencia, Spain; ^3^ Department of Physiology, Medicine School, University of Valencia, Valencia. Spain; ^4^ Sistemas genómicos, Valencia, Spain; ^5^ University of Valencia-UCIM, Spain; ^6^ Universidad de Las Villas, Las Villas, Cuba

**Keywords:** Werner Syndrome, Atypical Werner Syndrome, oxidative stress, antioxidant enzymes, thioredoxin, glutaredoxin, DNA damage, premature aging

## Abstract

Werner Syndrome (WS, ICD-10 E34.8, ORPHA902) and Atypical Werner Syndrome (AWS, ICD-10 E34.8, ORPHA79474) are very rare inherited syndromes characterized by premature aging. While approximately 90% of WS individuals have any of a range of mutations in the *WRN* gene, there exists a clinical subgroup in which the mutation occurs in the *LMNA/C* gene in heterozygosity. Although both syndromes exhibit an age-related pleiotropic phenotype, AWS manifests the onset of the disease during childhood, while major symptoms in WS appear between the ages of 20 and 30. To study the molecular mechanisms of progeroid diseases provides a useful insight into the normal aging process. Main changes found were the decrease in Cu/Zn and Mn SOD activities in the three cell lines. In AWS, both mRNA SOD and protein levels were also decreased. Catalase and glutathione peroxidases decrease, mainly in AWS. Glutaredoxin (Grx) and thioredoxin (Trx) protein expression was lower in the three progeroid cell lines. Grx and Trx were subjected to post-transcriptional regulation, because protein expression was reduced although mRNA levels were not greatly affected in WS.

## INTRODUCTION

Werner syndrome (WS, ICD-10 E34.8) or adult onset progeria is an uncommon, autosomal recessive human genetic disease that mimics premature aging. It is identified in the public portal of rare diseases as ORPHA902 (http://www.orpha.net) with a reported prevalence in worldwide population of 1/200,000 (Orphanet report). Due to the relatively non-specific nature of the symptoms and the lack of awareness of the condition, this disease may be under-diagnosed in many parts of the world [1].

Werner syndrome has been classified as a segmental progeria bearing some, but not all, aspects of aging. Its clinical phenotype has been succinctly summarized as a “caricature of aging” [2]. Patients with WS appear to age rapidly following puberty, developing graying and thinning hair, bilateral cataracts, osteoporosis, atherosclerosis, diabetes, and other age-related diseases.

This syndrome also confers a strong predisposition to several specific types of neoplasia [3]. Thus, the average life expectancy for WS patients was between 45-47 years, typically as a result of cancer or atherosclerotic cardiovascular diseases [4, 5], although over the last decade it has increased to more than 50 years of age in Japanese patients due to better clinical care and pharmacological interventions (use of pioglitazone and statins) [6].

The cells of these patients are prone to cellular senescence and neoplastic transformation and have an elevated genomic instability [7, 8] and sensibility to DNA damage induced by oxidative stress [4, 9].

The *WRN* gene, located on chromosome 8p12, encodes a 165 KDa multifunctional nuclear protein (WRN) that belongs to the conserved DNA RecQ family and possess both a 3' to 5' DNA helicase and a 3' to 5' DNA exonuclease domain [10, 11]. The RecQ-type helicase and exonuclease domains are situated in the central and N-terminal regions, respectively. Moreover, there is a nuclear localization signal at the C-terminal region and two consensus regions are located between the helicase and the nuclear localization signal (Supplementary Figure S1A). Both functions act in a coordinated manner in various DNA metabolic pathways, such as DNA repair, replication, and telomere maintenance correcting abnormal DNA structures generated after DNA repair, recombination and/or replication [12]. Thus, the WRN protein is implicated in the maintenance of genome stability [9, 13] and is deemed “caretaker of the genome” [4]. The importance of WRN-mediated pathway(s) for DNA repair and the replicational stress response has been demonstrated by measuring the levels of phosphorylated-gamma histone H2AX (γH2AX) due to acute loss of WRN which leads to DNA damage and high induction levels of γH2AX [14].

There have been approximately 70 mutations reported, both in coding and non-coding regions, for the WRN gene (International Registry of WS (http://www.wernersyndrome.org). The majority of these involve either a truncation of the protein or a shift in the reading frame [15]. The homozygotic null mutation of the WRN gene produces a shortening of the WRN protein, leading to the loss of its nuclear localization signal sequence. Thus the protein cannot be transported into the nucleus where it interacts with the DNA.

While approximately 90% of individuals presenting WS have any of a range of mutations in the *WRN* gene, [16] such as Q748X and F1074L mutations (Supplementary Figure S1A), a subset of patients shows some features of WS but they do not show mutations at the *WRN* locus and have normal levels and sizes of the WRN protein determined by Western blots [17]. Such progeroid syndrome was categorized as Atypical Werner Syndrome (AWS) (ORPHA79474) which refers to a heterogeneous group of cases that were clinically diagnosed as WS patients. These patients generally exhibit an age-related pleiotropic phenotype, characterized by short stature, thinning/graying hair, a “bird-like” facial appearance, skin atrophy, lipodystrophy, myopathy, osteoporosis, and atherosclerosis. When compared to WS, AWS patients appear to develop an earlier onset, more rapid rate of progression and possibly more severe age-related symptoms [18].

The AWS patients suffer the mutation of the lamin A/C gene (*LMNA/C*), the same causal gene seen in Hutchinson-Gilford progeria syndrome (HGPS) [19, 20]. Structural analyses have suggested the presence of missense and splicing mutations in *LMNA*-conserved residues [19] which principally affect exon 11 in this gene and correspond to the heptad repeat region of lamin A. Some substitutions do not change the amino acids but lead to the weak activation of the same cryptic splicing site, like in Hutchinson-Gilford progeria syndromes. By contrast, others like the E578V mutation, are thought to alter the interaction of lamin A with other proteins, or interfere with protein-protein interactions (Supplementary Figure S1B) [19, 20]. Therefore, the causes of AWS are molecularly heterogeneous and have not been fully elucidated [17].

Currently, no etiologic treatments for WS or AWS are available, only the clinical, age-related symptoms can be treated [5].

It is becoming increasingly evident that some of the molecular mechanisms of progeroid diseases might provide a useful insight into the normal aging process. This has been extensively reviewed by Dreesen and Stewart [21]. Conversely, processes linked to normal aging might be relevant to the pathogenic mechanisms of diseases, characterized by cancer predisposition or premature aging. Although aging is a complex and poorly understood process, a growing body of evidence points toward reactive oxygen species (ROS) as one of the primary determinants [22]. ROS have been identified as an important contributor to telomere shortening [23]. Notably, oxidative stress is a phenotypic hallmark in WS, AWS, Ataxia-Telangi-ectasia, Fanconi Anaemia, Down syndrome, and HGPS [24, 25]. The free radical theory of aging is not, however, the only theory proposed to explain the mechanism(s) involved in aging at the molecular level, but there are others, like telomere/cell senescence, genomic instability, and the mitochondrial hypothesis of aging [26].

Previous studies have investigated the role of the WRN protein in the cellular events contributing to senescence and age-related pathologies. More recent studies have been focused on evaluating the oxidative stress responses in WS. It has been observed that WRN depletion promotes a high induction of γH2AX and 53BP1 nuclear foci and accumulation of some prototypical oxidized bases, indicating a physiological function of WRN in oxidative damage repair in mammalian genomes [4, 9, 27].

AWS is fascinating from the standpoint of genetics, pathology, and aging but there is limited knowledge about its role in the maintenance of oxidative status. While some studies have evaluated the effect of progerin on the accumulation of oxidized proteins in fibroblasts from HGPS patients, the redox profile in AWS has never been explored.

In addition, the status of the major thiol redox systems in cells during aging and its modulation by the WRN protein has hardly been explored. Thus, the understanding of physiological and pathological processes involved in oxidative response, particularly associated with *WRN* and *LMNA* genes in these syndromes would provide insights into the mechanisms of natural aging and could give clues for developing strategies in which aging could be retarded.

Our ongoing study is aimed at evaluating the oxidative stress profile and antioxidant defense system of three cell lines from patients with different progeroid features. Specifically, we investigated how the expression and enzymatic activity of antioxidant systems are modulated by a heterozygous missense *LMNA* mutation, by a non-synonymous coding polymorphisms, and by a homozygotic null mutation in the *WRN* gene.

## RESULTS

### Fibroblasts from WS and AWS patients displayed low cell proliferation

The clinical features of patient donors of the cells are summarized in Table 1. All patients exhibited dermatological changes and other signs associated with aging that meet the International Registry Diagnostic criteria for the WS classification. Nevertheless, unlike patients with classic WS (WRN 1 and 2), who were diagnosed at around 30 years old, those diagnosed with AWS showed earlier and more aggressive symptoms, debuting at 13 years of age.

**Table 1:** Summary of clinical features of atypical Werner syndrome and Werner syndrome patients

Name	Code	Sex	Age	Syndrome/mutation	Clinical features
CTR1	GM01652	F	11		Healthy
CTR2	Donated	F	29		Healthy
CTR3	GM08402	M	32		Healthy
AWS	AG04110	F	13	AWS E578V LMNA	Accelerated aging. Short stature and dysmorphic features. Large coarse brown freckles and atrophic skin. Poor dentition and beak nose. Scoliosis. High-pitched voice.
WRN1	AG03141	F	30	WS Q748X WRN	Premature aging with pigmented and atrophic skin. Cataracts and hyperlipidemia type V.
WRN2	AG06300	M	37	WS F1074L WRN	Rapid onset of aging beginning at age 35. Muscle wasting and wrinkling of skin. High-pitched voice. Hypogonadism.

**Figure 1:**
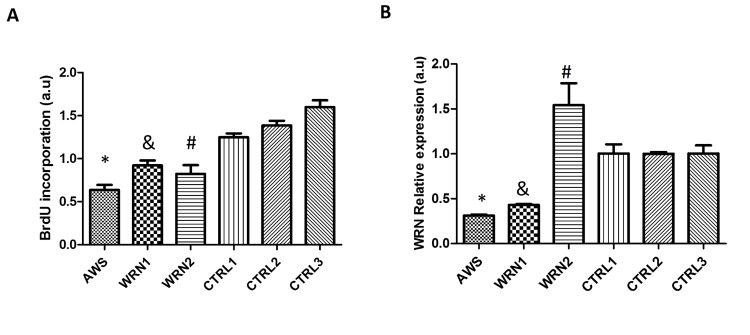
Characterization of atypical Werner Syndrome and Werner Syndrome fibroblasts (**A**) DNA synthesis in fibroblasts measured by the BrdU incorporation assay. Each WRN cell line was compared with its matched control in gender and age. (**B**) *WRN* mRNA levels measured during a proliferating phase, 48h after seeding the cells determined by RT-PCR, mean ±SD of three replicates. (*, &, # p <0.05).

To investigate the effects of different progeroid genotypes on fibroblast proliferation capacity, we evaluated bromodeoxyuridine (BrdU) incorporation in the three cell lines.Our results showed that all progeroid fibroblasts significantly reduced the BrdU incorporation 48h after cell culture compared with their matched controls (Figure 1A).

In addition, we assessed *WRN*mRNA levels in fibroblasts by using real-time PCR. The results obtained for the *WRN*gene expression were normalized using the levels of the control *GAPDH* mRNA. Low expression was observed for AWS and WRN1, unlike WRN2 that showed a higher expression compared to its matched control (Figure 1B).

### Increased oxidative stress in WS and AWS fibroblasts

**Figure 2:**
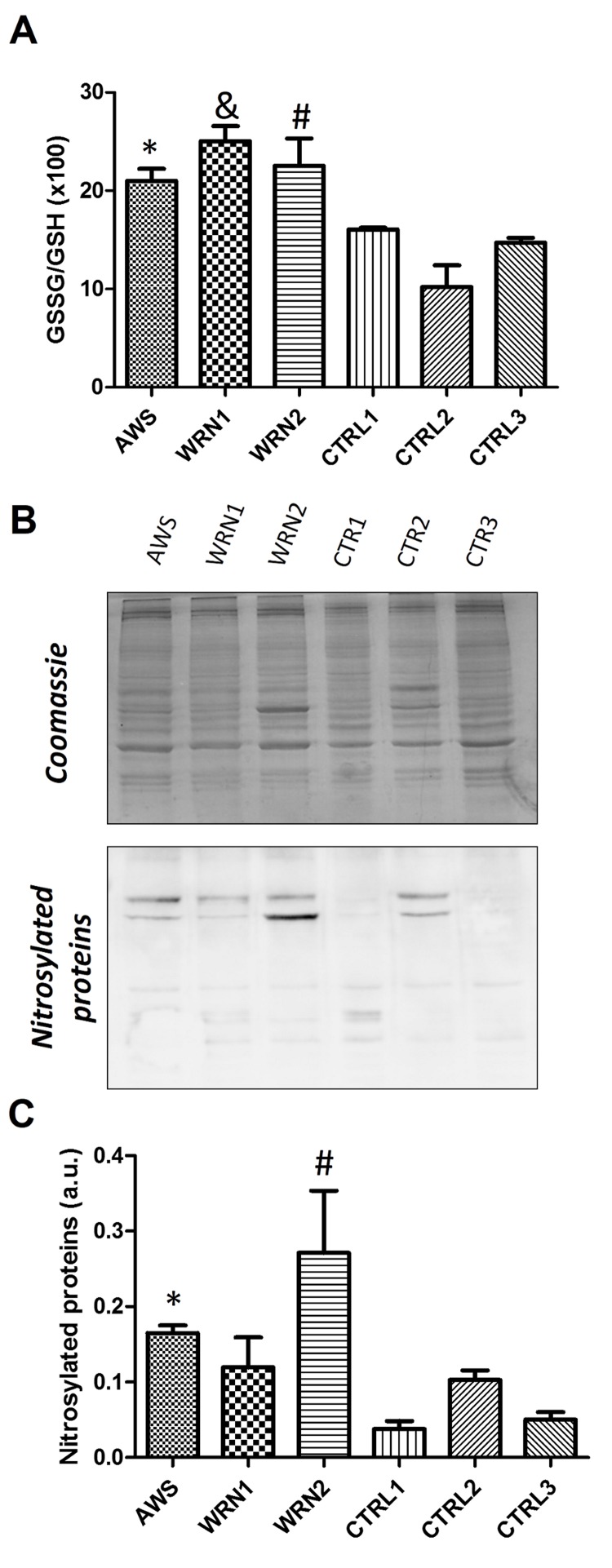
Analysis of the GSH antioxidant levels and Nitro-Tyr levels in atypical Werner syndrome and Werner syndrome fibroblasts (**A**) The GSSG/GSH ratio measured by triplicates. (**B**) Immunoblot of proteins from total extracts of AWS, WS, and control cells. (**C**) Densitometry of bands normalized by total protein amount using ImagJ software.

The ratio of reduced glutathione (GSH) to oxidized glutathione (GSSG) GSSG/GSH was measured to show the oxidative stress profile of WS and AWS fibroblasts (Figure 2A).

An increased GSSG/GSH ratio was observed for all progeroid fibroblast lines when compared with their matched controls; the highest ratio was observed in a WRN1 patient, indicating a more oxidative environment in this cell line.

In addition, we evaluated the cellular distribution of GSH by using the cell-permeant 5-chloromethyl-fluorescein diacetate (CMFDA) which is 95% specific for GSH [28]. As shown in Supplementary Figure S2A, fluorescence intensity was much lower in progeroid WRN cultures compared with their matched controls, indicating decreased GSH levels in the cytoplasm and nucleus of WRN cell lines. Furthermore, we analyzed the GSH compartmentalization within the cells. A tendency to low levels of nuclear GSH was found in the case of the AWS cell line compared with their counterpart (Control 1) (Figure S2B). However, when the WS cells were compared with their matched controls (Control 2) we observed low fluorescence and a high nuclear signal which indicates a GSH influx from the cytosol to the nucleus in WS, but not in AWS.

Tyrosinenitration is a common oxidative post-translational modification that affects protein structure and function and it is associated with pathogenesis in diseases related to oxidative stress in all living organisms. Tyrosinenitration is a biomarker of oxidative damage and protein molecular aging [29]. Thus, we evaluated the nitrotyrosine levels in proteins from the different cell lines. Our results indicate high levels of 3-nitrotyrosines in proteins obtained from WS and AWS cells, confirming an oxidative status in these cells obtained from patients (Figure 2B and 2C).

### Antioxidant defence in WS and AWS fibroblasts was downregulated

We examined the profile of antioxidant enzymes, which scavenge reactive oxygen species (ROS), in fibroblasts from progeroid cell lines. First, we studied mRNA levels for cytoplasmic and mitochondrial forms of superoxide dismutase (SOD1 and 2, respectively) as well as SOD protein content.

**Figure 3:**
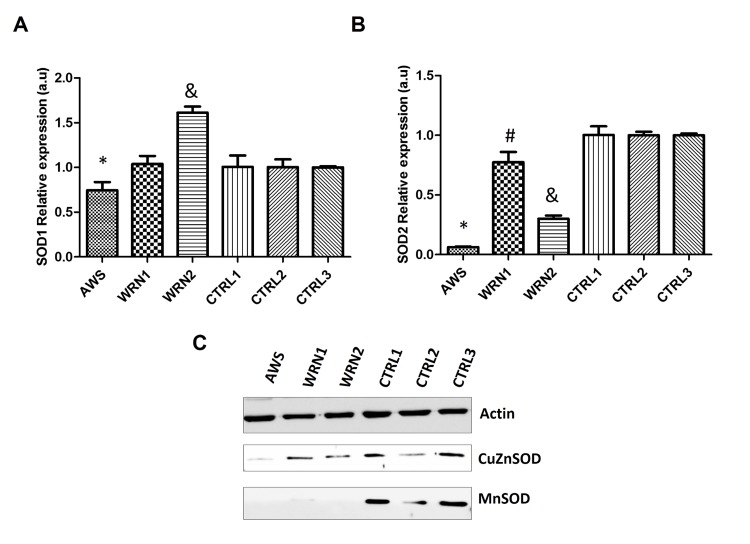
Analysis of the superoxide detoxifying enzymes in atypical Werner syndrome and Werner syndrome fibroblasts (**A**) Mean (±SD) mRNA levels of the SOD1 gene determined by qRT-PCR by triplicate. (**B**) Mean (±SD) mRNA levels of the SOD2 gene determined by qRT-PCR by triplicate. (**C**) CuZnSOD and MnSOD protein levels measured by Western blot in AWS, WS, and control fibroblasts.

Results shown in Figure 3A indicate a significant decrease in the *SOD1* gene for AWS. However, *SOD1* gene was overexpressed in the WRN2 cell line but no differences were observed between WRN1 and its matched control (Figure 3A). In the case of *SOD2*, it was significantly downregulated in the three cell lines and the lowest levels were detected in the AWS fibroblasts (Figure 3B).

Western blotting was used to study protein levels, but low levels of CuZnSOD were only identified in the AWS cell line (Figure 3C). We found low levels of the MnSOD protein for all three progeroid cell lines compared with their corresponding controls (Figure 3C). Therefore, the first step in ROS detoxification involved in the elimination of superoxide is impaired in AWS and WS cell lines.

**Figure 4:**
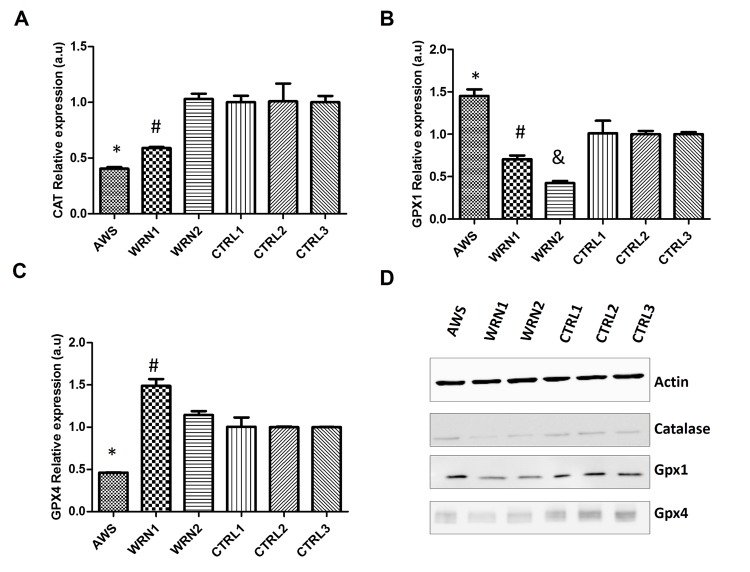
Analysis of the peroxide detoxifying enzymes in atypical Werner syndrome and Werner syndrome fibroblasts (**A**) Mean (±SD) mRNA levels of the *CAT* gene determined by qRT-PCR by triplicate. (**B**) Mean (±SD) mRNA levels of the *GPX1* gene determined by qRT-PCR by triplicate. (**C**) Mean (±SD) mRNA levels of the *GPX4* gene determined by qRT-PCR by triplicate. (**D**) CuZnSOD and MnSOD protein levels measured by Western blot in AWS, WS, and control fibroblasts.

The second step in ROS detoxification is the elimination of hydrogen peroxide and this reaction is performed mainly by catalase and glutathione peroxidases. Therefore, we analysed the expression of *CAT*,*GPX1,* and *GPX4* genes (which codify for the most active glutathione peroxidases). The analysis of the expression of these genes showed decreased levels for CAT mRNA for AWS and WRN1 compared with their controls (Figure 4A), but not for WRN2. The study of the *GPX1* gene expression indicated that it was upregulated in AWS cell lines. However, we detected a decreased expression for *GPX1* gene in the WRN1 and WRN2 cell lines from WS patients (Figure 4B). Nevertheless, the analysis of the *GPX4* expression showed downregulation for this gene only in AWS, but it was overexpressed in WRN1 (Figure 4C). The analysis of protein levels by immunoblot only showed differences for catalase in WRN1, but not in other cell lines compared with their paired controls. In addition, we detected differences in Gpx1 protein levels in WRN1 and WRN2 and their matched control cells (Figure 4D). Furthermore, we identified low protein levels of Gpx4 in all three cell lines compared with their control counterparts (Figure 4D), suggesting that the pathway for the reduction of oxidative damaged membrane lipids may also be affected in AWS and WS cells.

### Characterization of the main antioxidant enzyme activities in AWS and WS cells

**Figure 5:**
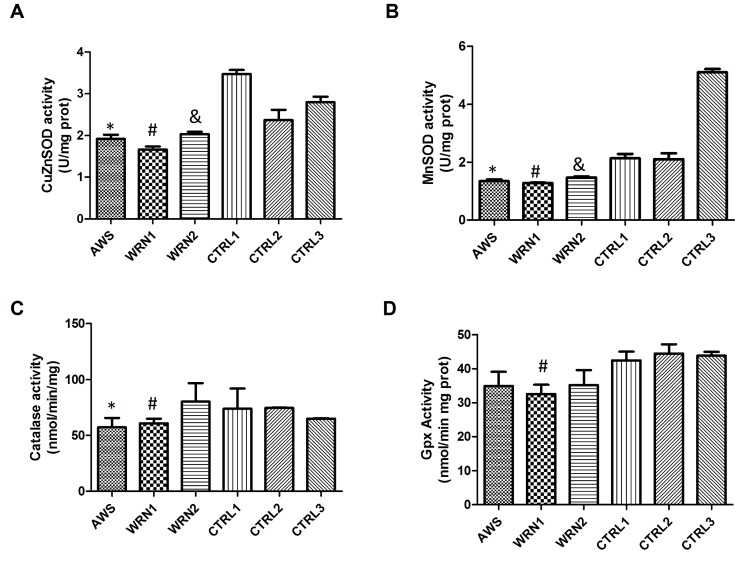
Analysis of the main antioxidant enzyme activities in atypical Werner syndrome and Werner syndrome fibroblasts (**A**) CuZnSOD activity and (**B**) MnSOD activity evaluated using the Superoxide dismutase assay kit (Cayman, Ann Arbor, MI, USA) based on reduction of the tetrazolium salt by superoxide to give rise the formazan which is measured at 460 nm. (**C**) Catalase activity was analysed spectrophoto-metrically after the formation of the purpald-formaldehyde adduct which is measured at 540 nm. (**D**) Total glutathione peroxidase activity determined by the Glutathione peroxidase assay kit (Cayman, Ann Arbor, MI, USA) based on the decrease of NADPH after coupling the glutathione reductase reaction that uses the NADPH to reduce the GSSG produced by the Gpx enzymes after reducing the cumene-hydroperoxide reagents. Results are represented as mean (±SD) of three replicates of each cell line.

To further characterize antioxidant responses in fibroblasts from AWS and WS, cells we determined the enzymatic activity of the main antioxidant systems studied. Superoxide dismutase activities, involved in the first step of ROS detoxification were studied by analyzing cytosolic (for CuZnSOD) and mitochondrial (for MnSOD) cellular extracts. The results indicated that CuZnSOD activity (Figure 5A) was significantly decreased in all progeroid cell lines when compared to their matched controls. Results obtained for MnSOD activity demonstrated that this activity was decreased in AWS and WS when the activity was compared with their control cell lines (Figure 5B). For catalase, the results shown in Figure 5C indicate that activities weredecreased in fibroblasts from the AWS and WRN1 cell lines compared to their age and gender matched controls, but not for WRN2. Finally, the analysis of total glutathione peroxidase activity (Figure 5D) showed no difference between fibroblasts obtained from AWS and WRN2 patients and their control cells. However, WRN1 showed significantly decreased activity. The results for total Gpx activity agree with those obtained for Gpx1 protein levels (the main contributor for the Gpx activity) in WRN1.

### Thioredoxin and glutaredoxin antioxidant defense is affected in AWS and WS

**Figure 6:**
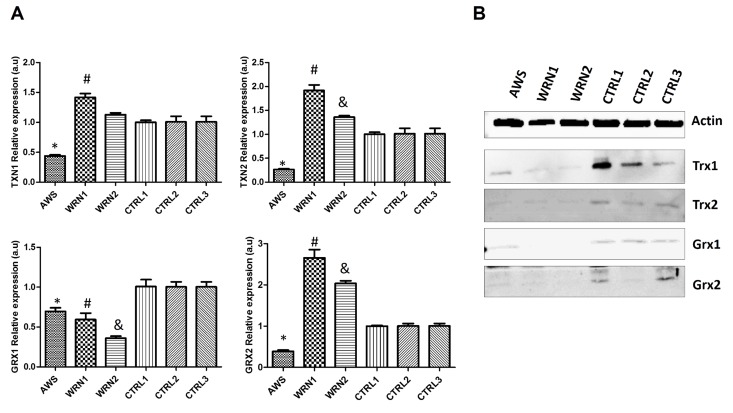
Analysis of the Trx and Grx family proteins in atypical Werner syndrome and Werner syndrome fibroblasts (**A**) Mean (±SD) mRNA levels of *TXN1, TXN2, GRX1, and GRX2* genes determined by qRT-PCR by triplicate. (**B**) Western blot analysis of the levels of Trx1, Trx2, and Trx3 antioxidant enzymes in fibroblasts from AWS, WS, and controls.

In order to complete the characterization of the antioxidant systems in WS and AWS fibroblasts, we analysed the levels of thioredoxins (Trx1 and Trx2) and glutaredoxins (Grx1 and Grx2). These proteins may be very relevant in these progeroid syndromes because they have an antioxidant function, they play a role in the elimination of disulphide bonds, they act as protein regulators, and they also participate in the synthesis of deoxyribonucleotides [30]. Thus, Trx and Grx are proteins that are directly involved in cell proliferation and also in DNA damage repair. We studied the expression of *TXN1*, *TXN2*,*GRX1,* and *GRX2* genes by qRT-PCR. We observed downregulation for the four genes in the cell line from AWS patient (Figure 6A). In addition, *GRX1* was downregulated in both WS cell lines. Nevertheless, *TXN1*, *TXN2,* and *GRX2* were overexpressed in both WS cell lines (WRN1 and WRN2) WRN2) (Figure 6A). Furthermore, we analysed the protein levels by Western blot. Our results indicate that the cytosolic and nuclear form Trx1 and the mitochondrial Trx2 protein levels were decreased in AWS and WS fibroblasts compared with their matched controls (Figure 6B). The study of cytosolic Grx1 and the mitochondrial and nuclear variant Grx2 levels indi- cates that these proteins were also decreased in AWS and WS cells (Figure 6B). These results suggest that Trx and Grx were subjected to post-transcriptional regulation, because protein expression was reduced although mRNA levels were not greatly affected in WS.

## DISCUSSION

Like Hutchinson-Gilford syndrome, Cockayne's syndrome, Xeroderma pigmentosum, and Down syndrome, the Werner syndrome is also deemed a segmental progeria in that it has some features, but not all aspects of aging [31]. Unlike other progerias, WS is considered an adult-onset progeria because of the symptoms that appear during puberty. In the specific case of atypical Werner syndrome, typical age-related disorders are referred to during puberty of the patient. Apart from the appearance of aged-related features, such as type II diabetes mellitus, osteoporosis, cardiovascular disease, cataracts, and dermal atrophy, other molecular age-related events contribute to portray the premature aging picture of the patients. The results shown in this work provide new clues to the molecular phenomena underlying the pathology of these very rare progeroid syndromes.

The contribution of ROS to aging was first suggested by the free radical theory of aging [32]. The role of ROS as the initial origin of aging is still controversial [26, 33], but it is clear that the accumulation of high levels of ROS during aging can damage tissues, cellular components, and biomolecules.

In addition, there is evidence of the involvement of oxidative stress *in vivo* in the phenotypes of progeroid syndromes, as previously reviewed by Pallardó *et al*. [34]. In views of these antecedents, we decided to study the molecular mechanism by which oxidative stress is a feature in progeroid syndromes using three cell lines with different genotypes and disease onset. The cell line from an AWS patient had the most severe phenotype [17].

Despite different genetic origins of each progeroid cell line, we observed decreased BrdU incorporation and hence cell proliferation was reduced in the AWS and WS cell lines compared with their matched controls (Figure 1A). It was reported that cells with an altered WRN protein may divide more slowly or stop dividing earlier than normal, causing growth problems [35, 36]. In the case of AWS, in which the mutation is in the *LMNA* gene, the altered capacity to proliferate may be due to aberrant conformation of the nuclear envelope. In fact, mutation E578V affects the interaction of the lamin A with other proteins and it also regulates *WRN* gene expression [17] and cell cycle progression [37].

The activity of mTOR (mammalian Target Of Rapamycin) is increased by oxidative stress [38]contributing to a senescent phenotype induced by an accumulation of progerin. Progerin stops the cell cycle by induction of mitotic/nuclear abnormalities [39]. When the cell cycle is arrested, as occurs in WS and AWS cells, mTOR can drive the senescent morphology. Thus, inhibition of the mTOR pathway is a strategy to decelerate aging in progerias [40]. Blagosklonny [41] has suggested rapamycin as a treatment for progerias and in fact it has reduced nuclear blebbing in HGPS fibroblasts [42].

Little information regarding changes in expression of the *WRN* gene determined by qRT-PCR analysis is available, but it is known that its levels are decreased in *in vitro* aged fibroblasts [43], sometimes by an augmented specific degradation of the*WRN* mRNA [44]. A number of authors agree that mutations, codifying for stop codons within the open reading frame, could target mutant *WRN* mRNA for non-sense mediated decay. In the particular case of the WRN1 cell line, which contains a Q748X mutation, mRNA stability is compromised. Furthermore,*WRN*polymorphisms may also affect *WRN*expression and function, although this has not as yet been rigorously examined [15, 45]. For the L1074F polymorphism (WRN2) the effect on *WRN* expression has still to be established. In our experiments, we observed overexpression of the *WRN* gene (Figure 1B). Previously, Marciniak *et al*. detected this protein as a wild type by immunoblot and immunofluorescence [46]. This polymorphism causes subtle changes in the helicase/exonuclease activities of the WRN protein [43]. We should keep in mind that F1074L is a common polymorphism found in the general population. The possibility of another unknown mutation (in another gene) in this patient and the contribution of epigenetic modifications during the life of the WRN2 patient should be considered. In addition, the role of the WRN protein in epigenetic control of pluripotent stem cell differentiation, could lead to Werner syndrome-like symptoms in this cell strain [47]. Furthermore, oxidative stress produces mTOR activation, which in turn increases progerin as discussed above, and also contributes to the senescence features including epigenetic aberrations [48]. Based on our findings and the changes in antioxidant proteins, WRN2 seems quite unique.

Glutathione (GSH) and thioredoxin (Trx) are two major thiol redox systems in animal cells during aging [49]. It is almost impossible to overstate the importance of GSH as the primary cellular antioxidant defense system. Moreover the increased GSSG/GSH ratio and changes in the GSH distribution, linked to increased nitrotyrosines in proteins reflects the oxidized redox state of the WS and AWS cells (Figure 2A, 2B and 2C), probably originated by an impairment of metabolic pathways or mitochondrial dysfunction as indicated previously [34], and also by a defective antioxidant enzymatic shield. Furthermore, manifestations of WS may reflect the impaired ability of slowly dividing cells to control oxidative DNA damage [50].

The analysis of the main antioxidant systems in the three cell lines points out defective antioxidant enzymatic function. A good correlation between gene expression and protein levels in AWS was found (Figure 3 and 4), indicating low levels and activity (Figure 5) of the main antioxidant enzymes in the AWS cell line . Our results agree with those obtained by Yan *et al.* for HGPS cells which also possesses mutations in *LMNA/C* gene [51], mainly for MnSOD, catalase and Gpx. However, our results did not correlate with those described by Viteri *et al*. for HGPS cells [25], where the main antioxidant enzymes were overexpressed. Differences between the results in the expression of the antioxidant enzymes could be due to the different mutations in *LMNA* genes in the different studied cell lines, the heterozygosis of AWS, or the method of protein extraction.

We observed a different scenario between both the WRN cell lines (WRN1 and WRN2), probably linked to the different mutations of origin. WRN1 possesses a mutation that produces a truncated WRN protein, while a replacement of amino acid in the DNA binding site of WRN protein occurs in WRN2. Gene expression and protein levels did not correlate completely (Figure 3 and 4), suggesting post-transcriptional regulatory control. The analysis of protein levels showed differences in the main peroxide detoxifying enzymes (catalase, glutathione peroxidase 1, and glutathione peroxidase 4) in WRN1, suggesting that this second step is impaired in the patient in whom the WRN protein is truncated (Figure 5D) [52, 53]. After analysis of the antioxidant enzyme activity, WRN1 showed all the antioxidant activities that were affected compared with WRN2, which only showed deregulation in SOD activities (Figure 5), pointing out that truncation of WRN protein is associated with a more aggressive phenotype. Furthermore, Trx and Grx systems were seriously decreased in WRN fibroblasts compared with control cells (figure 6). Therefore, our results point out that Trx and Grx proteins are directly involved in the physiopathology of these progeroid syndromes, contributing to decreased antioxidant defense and low proliferation capacity of these cells. A deleterious effect of this defect is that these patients' wounds heal with difficulty and their recovery after surgical intervention, as Walton *et al*.reported [54]. Interestingly, down-regulation of Trx2 produces impairment in the mitochondrial function and induces increased oxidative stress in mice [55]. Notably, the implication of the Trx and Grx protein family has not been completely elucidated in progeroid syndromes, although Trx proteins have a relevant role during aging [49]. Pioneer work by Cho *et al*. showed low levels of Trx in tissue from old rats [49]. Our results in WS and AWS cells underscore the notion that downregulation of Trx proteins is involved in the features of aging and progeroid syndromes.

In conclusion, our results showing low levels of MnSOD, Trx2, and Grx1 proteins direct our attention to mitochondria in both WS and AWS. Thus, mitochondria are a target in the physiopathology of WS and AWS, as suggested for other progeroid syndromes [34]. However, we detected clear differences in the origin of the disability of the antioxidant enzymes occurring in the three cell lines. The origin of the antioxidant enzyme deregulation has a transcriptional origin in the AWS cell line. This result suggests the implication of chromatin structure and its regulation [56]. On the other hand, the major clinical features described in WS patients are produced as a consequence of absolute lack of the normal WRN protein in the nucleus, which produces DNA replication dysfunction [57], DNA repair deficits [58], transcription deregulation [59], and deregulation of chromatin structure [60]. Therefore, although oxidative stress is observed in both the WRN1 and WRN2 cell lines, deficient antioxidant enzyme activity is marked in WRN1, which correlates with a more aggressive phenotype in this patient compared to WRN2. In sum, oxidative stress and low antioxidant defense contributes to the severe phenotype of AWS and also in the WRN1 with a truncated protein, indicating that oxidative stress is closely associated with progeroid features in these genetic instability disorders.

## METHODS

### Cell culture

Threehuman fibroblast cell strains AG04110, AG03141, and AG06300, selected based on the search terms “progeroid phenotype”, were acquired from Coriell Cell Repositories (Camden, NJ, USA http://locus.umdnj.edu/ccr/) from donors matched for gender, similar ages, and similar passage levels. In our work were denominated as AWS, WRN1 and WRN2, respectively.

Out of three cell lines (AWS, AG04110) corresponds to AWS, bearing a known missense heterozygous mutation (E578V) in exon 11 of *LMNA* gene (Supplementary Figure S1B) [61]. Patients corresponding to cell lines AG03141, and AG06300 were diagnosed with WS. WRN1 (AG03141) is homozygous for a C to T transition at nucleotide 2476 in the *WRN* gene (2476C>T), corresponding with exon 19 of helicase domain and resulting in a stop codon at 748 (Q748X) (Figure 1A) [62]. Cell line AG06300 (WRN2) carries a nucleotide change (3456T/G exon 26) in the helicase RNaseD C-terminal conserved region of WRN gene (refSNP ID: rs2725362), resulting in a non-synonymous coding polymorphisms (F1074L) but with expression of normal amounts of full-length WRN protein, in the same sub-cellular domains [46].

The cells were cultured in Eagle's minimum essential medium with Earle's salts and non-essential amino acids (DMEM, Gibco, Invitrogen) supplemented with 15% fetal bovine serum inactivated, and 1% penicillin-streptomycin (Sigma-Aldrich, St. Louis, MO) in 5% CO_2_ in air at 37°C and density of 20,000 cells/cm^2^. Trypsin-EDTA was used as the subculture method. Studies were performed at cell confluence with the exception of BrdU incorporation assay and GSH cellular distribution analysis by confocal microscopy.

### Cell proliferation studies by BrdU incorporation assay

Proliferation of cell lines was determined with the “Cell proliferation ELISA BdrU colorimetric assay” from Roche. Cells were cultured in 96 wells plates at density of 20,000 cells/ cm^2^ during 48 h and 6 h before absorbance reading BrdU was incorporated to the culture medium. Afterwards, ELISA assay was performed following the manufacturer's protocol. The final reaction was measured with the spectrophotometer Spectra MAXPLUS 384 from Molecular Devices, at wavelengths of 370-492 nm and 3 intervals of 5 min.

### GSH/GSSG measurements

2·10^5^ cells for each cell type were lysed in cold 5-sulfo-salicylic acid dehydrate at 5% weight/volume. Then, cell suspension was centrifuged at 14,000 xg at 4°C during 10 min. Afterwards, supernatants were used to measure free GSH and total GSH, by Glutathione reductase, and NADPH coupled reaction following the manufactures instructions of “DetectX, Glutathione Fluorescent Detection kit” from ArborAssays (Ann Arbor, Michigan USA). Fluorescence readings were performed using fluorescent emission at 510 nm with excitation at 390 nm using a fluorimeter spectraMAX GEMINI (Molecular Devices, Sunnyvale, USA). GSSG levels were calculated using the formula GSSG = (Total GSH- Free GSH)/2.

### Confocal microscopy

AWS and control fibroblasts were cultured as described above in 2 cm^2^ Lab-Tek II chambered cover glass (Nunc) for 48h. One hour before the measurement of GSH distribution double staining was performed maintaining cells alive. First, 5 μM Cell Tracker green 5-chloromethylfluorescein diacetate (CMFDA) (Molecular Probes) to detect GSH in the cells was added to the cell culture medium for 30 min at 37°C and 5% CO_2_. After washing with cell culture medium, cells were left for additional 30 min of incubation. Afterwards, cells were stained using 2 μg/mL Hoechst 33342 (Sigma-Aldrich) to localize nuclei. Two additional washes using cell culture medium was performed and then GSH cellular distribution images were acquired using a Leica TCS-SP2 confocal laser scanning unit equipped with argon and helium-neon laser beams and attached to a Leica DM1RB inverted microscope. The excitation wavelengths for fluorochromes were 488 nm for CMFDA and 364 for Hoechst. The emission fluorescence was detected at 510-540 nm for CMFDA and 380-485 for Hoechst. Maximum projection was acquired at least in five different fields. The quantification of the fluorescence in each cellular compartment was obtained using ImageJ. Perimeters were drawn around the nucleus (according to the area marked with Hoechst) and around the entire cell (according to the area marked with CMFDA). The nucleus/total ratio and cytoplasm/total ratio for GSH in every cell analyzed was established by dividing the mean of green CMFDA fluorescence of nuclear or cytoplasm area by the mean of CMFDA fluorescence in total area.

### qRT-PCR

Total RNA was isolated from cells using the PARIS™ Protein and RNA Isolation System (Ambion, Austin, TX) according to the manufacturer's instructions. For reverse transcription reactions (RT), 1 μg of the purified RNA was reverse transcribed using random hexamers with the High-Capacity cDNA Archive kit (Applied Biosystems, P/N: 4322171; Foster City, CA) according to the manufacturer's instructions. RT conditions comprised an initial incubation step at 25°C for 10 min to allow random hexamers annealing, followed by cDNA synthesis at 37°C for 120 min, and a final inactivation step for 5 min. at 95°C.

The mRNA levels were determined by quantitative real-time PCR analysis using an ABI Prism 7900 HT Fast Real-Time PCR System (Applied Biosystems, Foster City, CA). Gene-specific primer pairs and probes for *WRN (WernerHs01087915_m1, Taqman® Assays, Applied Biosystems)*, *SOD1* (*SOD Cu/Zn Hs00533490_m1, Taqman® Assays, Applied Biosystems)*, *SOD2* (*SOD Mn Hs00167309_m1, Taqman® Assays, Applied Biosystems)*, *GPX1 (Glutathione peroxidase 1 Hs00829989_gH, Taqman® Assays, Applied Biosystems)*, *GPX4 (Glutathione peroxidase 4Hs00989766_g1, Taqman® Assays, Applied Biosystems), CAT* (*CatalaseHs00989766_g1, Taqman® Assays, Applied Biosystems), TXN1 (Thioredoxin 1Hs01555214_g1, Taqman® Assays, Applied Biosystems) , TXN2 (Thioredoxin 2 Hs00912509_g1, Taqman® Assays, Applied Biosystems), GRX1 (Glutaredoxin 1 Hs00829752_g1, Taqman® Assays, Applied Biosystems), and GRX2 (Glutaredoxin 2 Hs00375015_m1, Taqman® Assays, Applied Biosystems)*Biosystems), were used together with 1x TaqMan® Universal PCR Master Mix (Applied Biosystems, P/N 4304437, Foster City, CA) and 2 μl of reverse transcribed sample RNA in 20 μl reaction volumes. PCR conditions were 10 min. at 95°C for enzyme activation, followed by 40 two-step cycles (15 sec at 95°C; 1 min at 60°C). The levels of glyceraldehyde-3-phosphate dehydrogenase (*GAPDH Hs00375015_m1, Taqman® Assays, Applied Biosystems*) expression were measured in all samples to normalize gene expression for sample-to-sample differences in RNA input, RNA quality and reverse transcription efficiency. Each sample was analysed in triplicate, and the expression was calculated according to the 2^−^^ΔΔ^^Ct^ method .

### Protein levels by Western blot

Proteins in the sample were denatured using the samplebuffer (Tris 40mM, EDTA, bromophenol blue 0,01%, sucrose 40%, SDS 4%, β-mercaptoethanol 10%)and heating to 95°C for 5 minutes. The samples were electrophoresed in a 12% SDS-PAGE. After that, the proteins were transferred onto Nitrocellulose membrane (Whatman GmbH, Dassel, Germany).

After transference the membrane was blocked with milk 5% in TBS-Tween for 1 hour. Afterwards, membrane slice depending on the molecular weight of the protein of interest were incubated with specific monoclonal antibodies. Catalase (1:1000) (Sigma, St. Louis, USA), MnSOD (1:1000) (Stressgen, Ann Arbor, MI, USA), CuZnSOD (1:1000, Stressgen, Ann Arbor, MI, USA), Gpx1 and Gpx4 (1:750, Abcam, Cambridge, MA, USA), Trx1 (1:1000, Abcam), Trx2, Grx1 (1:1000, Abcam), Grx2 (1:1000, Abcam). As loading controls, antibodies that recognize α-tubulin or β-actin (1:1000, Santa Cruz BioTech. USA) antibodies were used. Thereafter, the blots were washedagain with TBST and further incubated for 1 h with a secondary mouse, rabbit or goat antibody conjugated withhorseradish peroxidase-linked. The membrane was incubated for 50 minutes at room temperature and constant agitation. Finally, the membrane was washed 3x5 minutes with TBS-TWEEN. Chemioluminiscent reagent was added onto the membrane (Luminol: ECL Western Blotting Detection Reagents, GE Healthcare, UK) 1 ml/membrane, and membranes were revealed by an image reader LAS-4000) (General Electrics Healthcare). Results were then analysed with Image software 4.0 Gauge.

### Antioxidant enzymes activity

Measurement of antioxidant activities were performed as previously described in García-Giménez *et al.*[63]. For CuZnSOD and MnSOD the Cayman “Superoxide Dismutase assay kit” (Cayman, An Arbor, MI) and for the measurement of total Gpx activity “Glutathione peroxidase assay kit (Cayman, An Arbor, MI) were used. For the measurement of catalase enzymatic activity the method was based on the reaction of the enzyme with methanol in the presence of hydrogen peroxide to produce formaldehyde. All spectrophotometric measurements were performed using a spectrophotometer Spectra MAXPLUS384 (Molecular Devices, Sunnyvale, CA, USA).

### Statistical analyses

For the statistical analysis of the results, the mean was taken as the measurement of the main tendency, while standard deviation was taken as the dispersion measurement. T-student test was performed to compare differences between WRN cell lines and their matched controls analyzing GSSG/GSH ratio, and nitro-Tyrosine protein levels, expression of *WRN*, *SOD1*, *SOD2*, *CAT*, *GPX1*, *GPX4, TRX1, TRX2, GRX1, and GRX2* genes and activities for CuZnSOD, MnSOD, catalase and glutathione peroxidase. Different number of technical replicates was used in each analytical determination (see specific technique for details). The alpha level for statistical significance was set at p < 0.05.

## SUPPLEMENTAL FIGURES


