# Supplementary material for: Oxidative stress and antioxidant response in fibroblasts from Werner and Atypical Werner Syndromes
Source: Aging (Albany NY). 2014 Mar 31;6(3):231–45. doi: 10.18632/aging.100649 (PMC4012939; doi:10.18632/aging.100649)
Supplement: Supplementary file 1 [file aging-06-231-s001.pdf]

SUPPLEMENTARY FIGURES

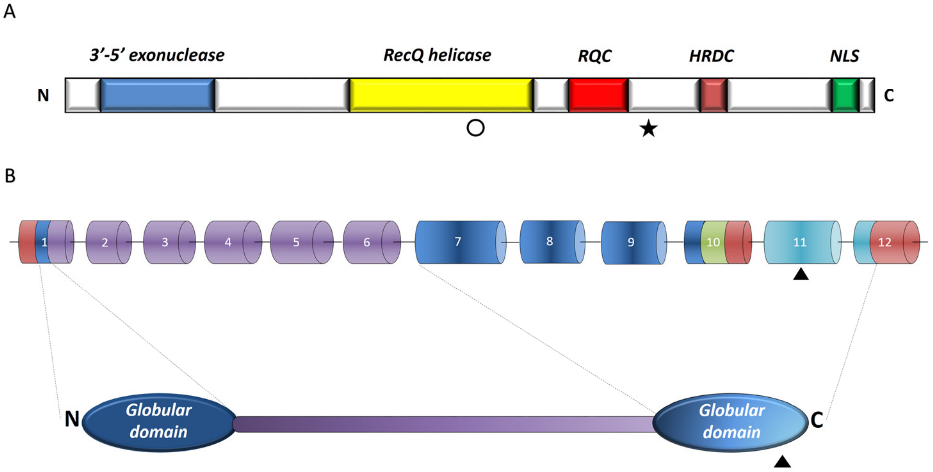

Supplementary Figure 1. Scheme for the mutations in A) the WRN gene for Werner Syndrome (WS) and B) in the *LMNA* gene for atypical Werner Syndrome (AWS).

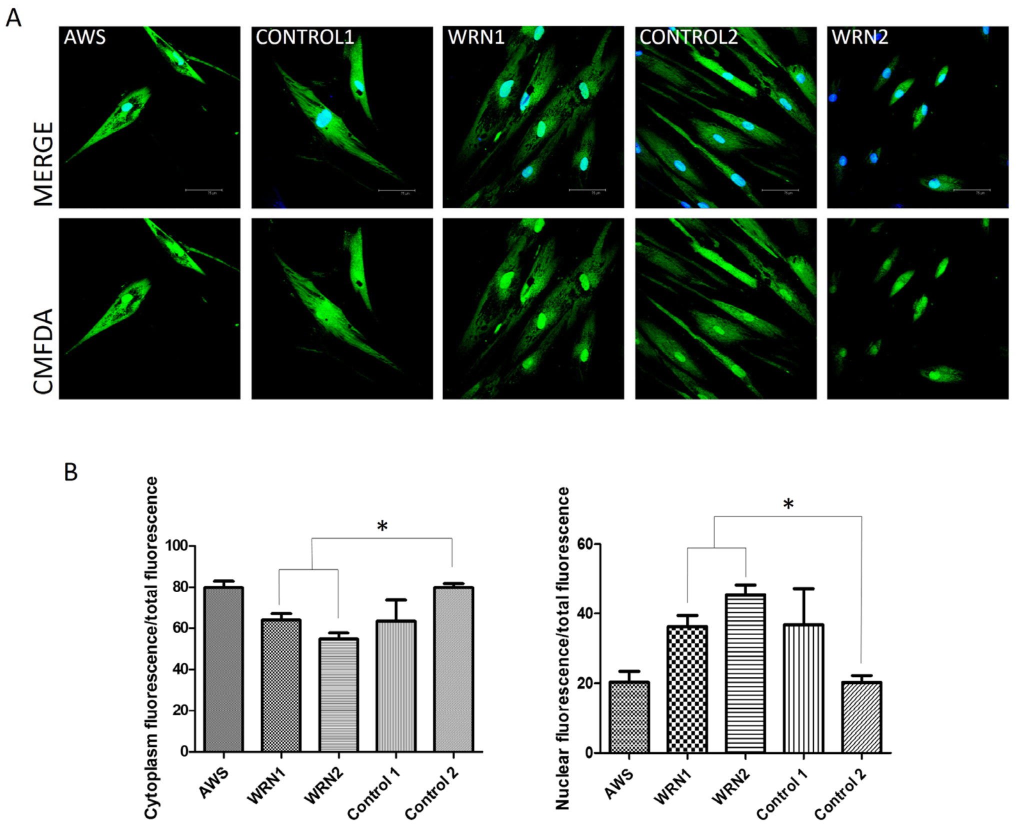

Supplementary Figure 2. Cellular GSH distribution in fibroblasts measured by confocal microscopy. (**A**) Green indicates distribution of GSH in the cellular compartments stained with 5-chloromethylfluorescein diacetate (CMFDA). Blue indicates the nucleus stained with Hoechst. The emission fluorescence was detected at 510-540 nm for CMFDA and 380-485 for Hoechst. Maximum projection was acquired in at least five different fields. (**B**) Fluorescence quantification of CMFDA dye in the nucleus and cytoplasm in AWS and WS cells. Results are represented as mean (±SD) of 20 cells counted at least in 3 different experiments.
